# Digital-PCR for gene expression: impact from inherent tissue RNA degradation

**DOI:** 10.1038/s41598-017-17619-0

**Published:** 2017-12-08

**Authors:** Melanie J. Millier, Lisa K. Stamp, Paul A. Hessian

**Affiliations:** 1 University of Otago, Department of Medicine, P.O. Box 56, Dunedin, 9054 New Zealand; 2 University of Otago Christchurch, Department of Medicine, P.O. Box 4345, Christchurch, 8140 New Zealand

## Abstract

Subtle molecular differences indicate the heterogeneity present in a number of disease settings. Digital-PCR (dPCR) platforms achieve the necessary levels of sensitivity and accuracy over standard quantitative RT-PCR (qPCR) that promote their use for such situations, detecting low abundance transcript and subtle changes from gene expression. An underlying requisite is good quality RNA, principally dictated by appropriate tissue handling and RNA extraction. Here we consider the application of dPCR to measures of gene expression in pathological tissues with inherent necrosis, focusing on rheumatoid subcutaneous nodules. Variable RNA fragmentation is a feature of RNA from such tissues. Increased presence of transcript fragmentation is reflected in a proportionate decrease in Agilent DV_200_ metric and downstream, a reduction in endogenous control genes’ expression, measured by RT-dPCR. We show that normalisation of target gene expression to that for endogenous control genes sufficiently corrects for the variable level of fragmented RNA. Recovery of target gene values was achieved in samples comprising as much as 50 percent fragmented RNA, indicating the suitability and appropriate limitation of such data treatment when applied to samples obtained from inherently necrotic tissues.

## Introduction

Digital PCR (dPCR) is becoming a valuable technique for the quantification of gene expression, providing increased sensitivity, reproducibility and linearity when compared to real-time quantitative PCR (qPCR). These advantages are particularly relevant for low-abundant targets^1–3^.

Recent studies have shown that reverse transcription dPCR (RT-dPCR) technology can overcome the adverse impact of a range of background sample contaminants and PCR inhibitors. This enables the generation of reliable data that, in some cases, may not require normalisation to reference genes^1^. However, there is currently little information available specifically concerning the rationale and efficacy of reference gene normalisation to correct for variable quality of input RNA in RT-dPCR. Such variation is more likely when sampling from disease-associated or tumour tissues that incorporate inherent necrosis or where significant areas are affected by mechanisms leading to cell death^4–6^. Under such conditions, the degradation of cellular RNA can be up-regulated by specific signalling pathways^7–9^, or as a consequence of therapeutic drug response^10^.

The pitfalls of using low quality mRNA in quantitative molecular analyses are well-established^11–13^. Studies that caution the use of degraded RNA in downstream applications highlight the characteristically unstable nature of mRNA and its susceptibility to RNA hydrolases (RNases), particularly during the acquisition, handling and storage of tissue samples. Transcript-specific degradation has also been described as a consequence of delayed post-excision or post-mortem RNA preservation^11–13^. However, the integral quality of resource tissue, particularly of valuable human pathological samples, provides an equally important barrier to molecular analysis. The underlying challenge researchers face when working with pathological, often rare, study samples is the acquisition of quality, biologically meaningful data from an adequate number of samples^4^.

A focus of our investigations into chronic inflammation has concerned pathogenic mechanisms within the rheumatoid subcutaneous nodule^14^, a granulomatous lesion generally associated with more severe rheumatoid arthritis. A feature of rheumatoid nodules is the “natural” presence of areas of necrotic tissue, accompanied by varying degrees of cellular apoptosis, both of which are an inherent part of the inflammatory process^5,15^. RNA from these areas is unavoidably extracted and consequently present as a variable component of nodule tissue RNA used for downstream studies. Fragmented RNA of at least 100 nucleotides in length contributes to the total RNA concentration measurement prior to RT-dPCR^16^, although because many transcripts may not be sufficiently intact for complete reverse transcription, correction is required to ensure that the final data reflects gene expression in functional, non-necrotic areas of the tissue.

Here we consider the application of nanofluidic partitioning chip-based dPCR to quantitative gene expression in tissues complicated by necrosis. We sought to utilise this technique to exploit the increased detection sensitivity and precision capabilities attributed to dPCR, when compared to qPCR, while maintaining an existing, well-validated, sample processing workflow. Results show that measures of the expression of appropriate endogenous control genes, like GAPDH and HPRT1, are proportionate to the level of mRNA of quality sufficient to allow reliable reverse-transcription. We also sought to establish suitable inclusion criteria for samples according to the accuracy of normalisation. This approach limits the undesirable impact on target gene expression values that can arise when working with tissues containing inherent necrosis, while still maintaining the high level of detection sensitivity of the digital technology.

## Results

### RNA fragmentation

Retrospective histological analysis showed that all nodule tissues used for RNA extraction contained palisading monocyte/macrophages, focused on a central necrotic area (data not shown). This is a characteristic feature of granulomatous rheumatoid nodules, that we have previously described^5,17^.

**Figure 1:**
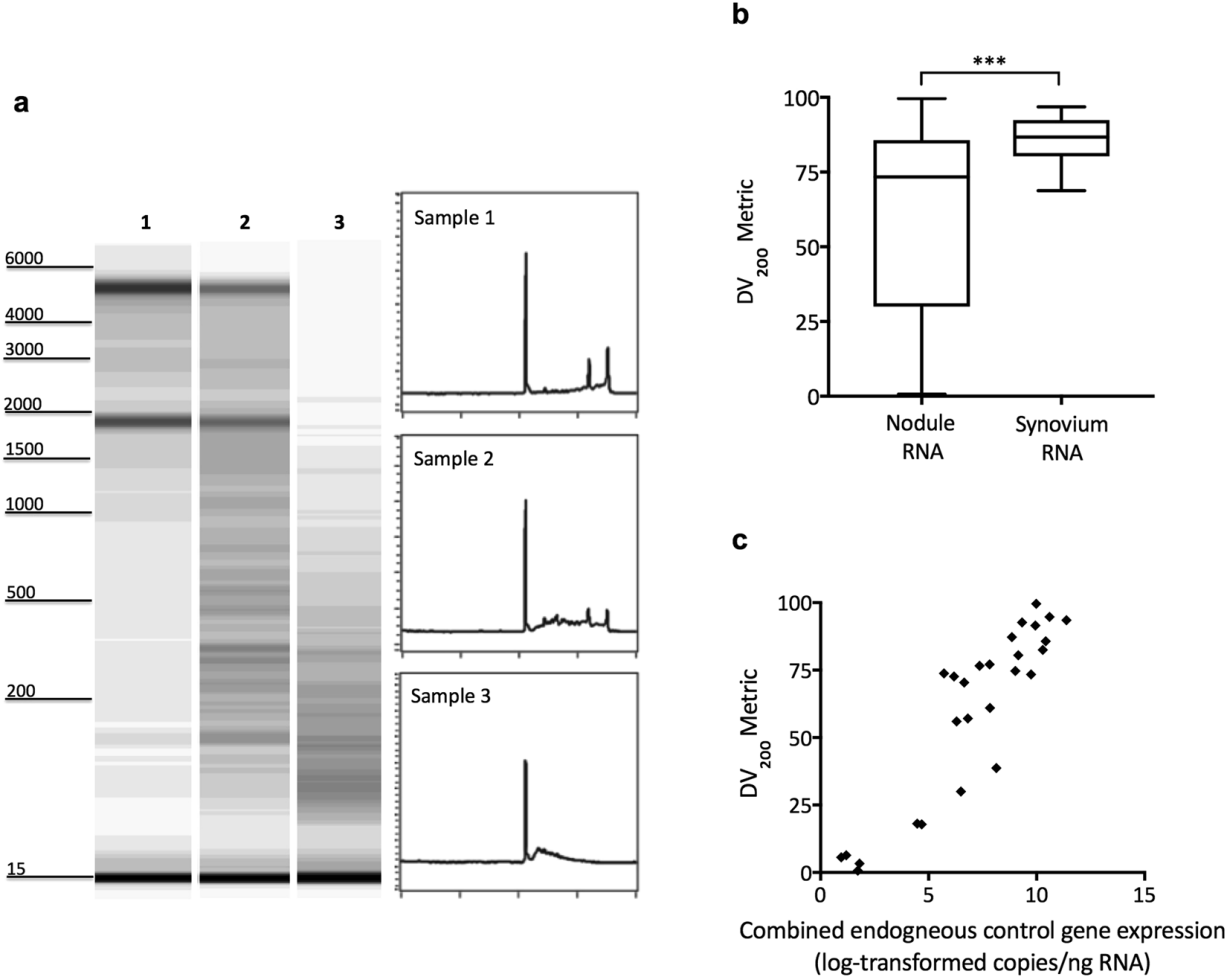
RNA quality analysis and gene expression. (**a**) Capillary electrophoresis gel images and electropherograms show the range of RNA quality from representative nodule tissues (1–3). Corresponding DV_200_ metric values (and accompanying RNA Quality Number, RQN) were: sample 1 = 93.5%, (7.4); sample 2 = 80.5%, (3.3); sample 3 = 38.7%, (1.0). (**b**) Tukey box and whiskers graph displaying average DV_200_ metrics for either nodule or synovial membrane RNA samples, which undergo identical sample handling; ***p = 0.0005. (**c**) Sample DV_200_ metric values for nodule-derived RNA are positively correlated with the levels of combined endogenous control gene (GAPDH and HPRT1) expression (r = 0.9087; *p* < 0.0001). Capillary electrophoresis gel images shown in (**a**) for samples 1–3 are from an original results figure (pdf file), contrast-enhanced to highlight fragmented RNA; Each image was then individually cropped and uniformly resized. Electropherograms shown are black and white versions from an original results figure (pdf file) with no further manipulation. Full versions of these original figures are presented in Supplementary Figures S1 and S2.

RNA quality analysis revealed that RNA purified from nodule tissues was variably fragmented between samples, despite consistent and best practice for sample handling and RNA preservation (Fig. 1a). The extent of fragmentation in each sample was quantitatively expressed by using the DV_200_ metric, a measure of the percentage of RNA transcripts >200 nucleotides in length. We compared the DV_200_ metrics measured in nodule RNA samples with those in synovial membrane RNA, finding greater variability in RNA fragmentation in nodules (Fig. 1b). Consequently, synovial RNA samples contain a significantly higher proportion of longer RNA transcripts (*p* = 0.0005). Both tissue types undergo similar handling suggesting that the quality of nodule RNA reflects *in vivo* cellular processes characteristic of these pathological, necrotic lesions.

Finally, we assessed GAPDH and HPRT1 expression in nodule samples and found a significant positive correlation (r = 0.9087, *p* < 0.0001) between combined expression levels for these two endogenous control genes and the DV_200_ metric (Fig. 1c).

### Validation study

**Figure 2:**
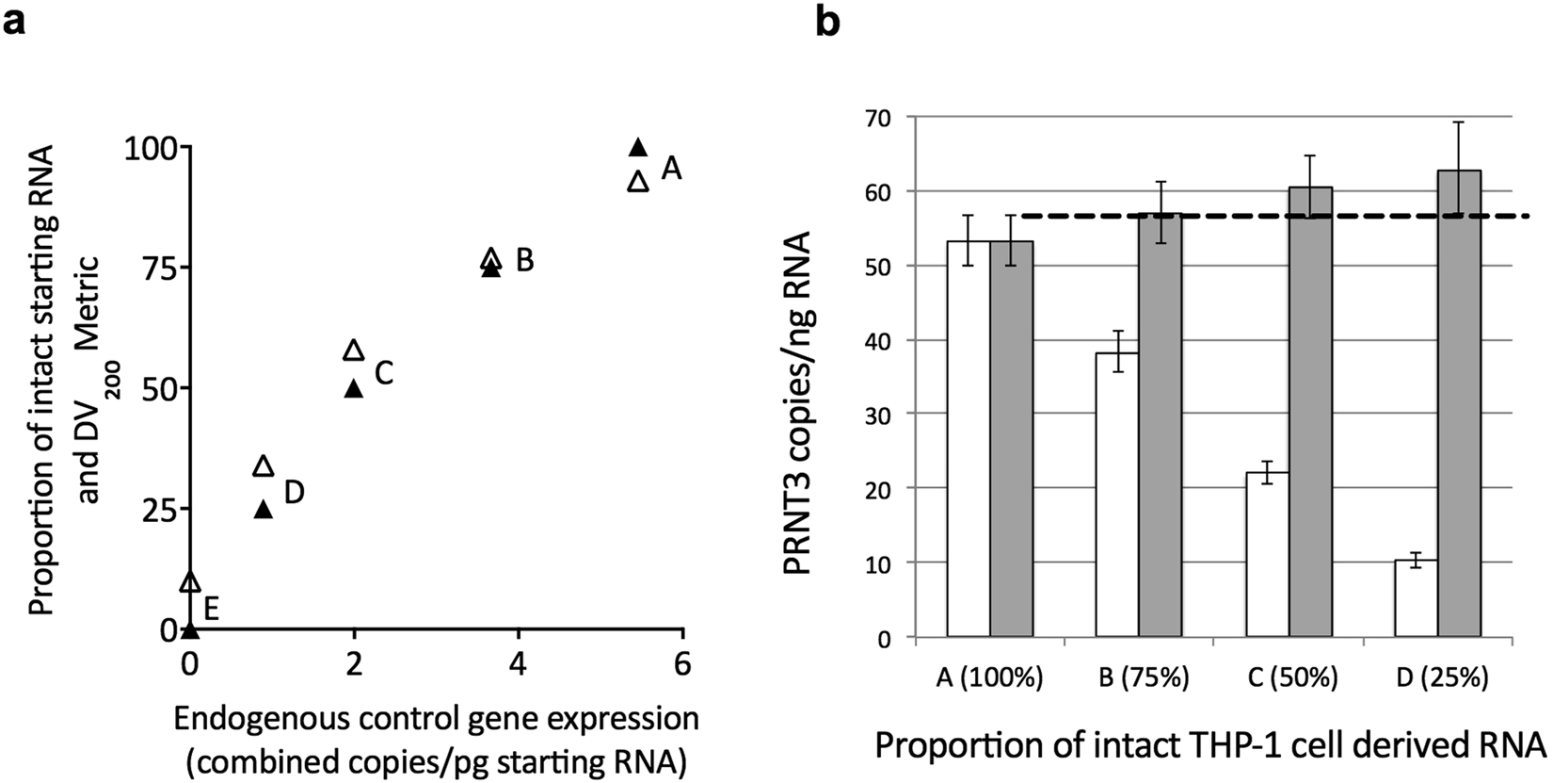
Influence of fragmented RNA and data normalisation on gene expression. (**a**) A series of RNA samples (A–E) containing set proportions of intact THP-1 cell RNA, spiked with increasing proportions of highly-fragmented nodule RNA. Corresponding RNA integrity number (RIN) values for the samples were: A: 8.3; B: 8.5; C: 8.1; D: 4.6; E: 2.5. Using dPCR, combined and averaged absolute measures of GAPDH and HPRT1 endogenous control gene expression positively correlate with both the proportion of pre-RT intact RNA (▴; r = 0.9891; *p* = 0.0014) and the DV_200_ metrics (Δ; r = 0.9729; *p* = 0.0053). (**b**) Absolute (▫) and normalised values () of PRTN3 expression in samples (A–D), with the 95% CI for the copies/pg RNA. The dashed line marks the upper 95% CI limit for sample A. Normalisation was applied according to the calculated endogenous control gene normalisation factors of A: 1; B: 1.49; C: 2.74; D: 6.12; E (not displayed): 3232.

To investigate the application of normalisation to dPCR data, we generated samples of intact THP-1 cell-derived RNA spiked with known proportions of naturally, highly fragmented nodule RNA. As expected, the DV_200_ metrics closely paralleled the proportion of spiked, fragmented RNA present in each of these samples (Fig. 2a). Expression of the endogenous control genes, GAPDH and HPRT1, significantly correlated with the DV_200_ metrics (r = 0.9729; *p* = 0.0053) and with the proportion of intact starting RNA (r = 0.9891, *p = *0.0014) (Fig. 2a). Interestingly, only when the proportion of intact RNA within spiked samples fell below 50 percent was there a noticeable drop in RNA Integrity Number (RIN) values (detailed in Fig. 2a legend).

RNA-seq data indicated low or undetectable expression of proteinase 3 (PRTN3) in nodules *per se*. Therefore, irrespective of quality, nodule RNA is not expected to contain transcript that might significantly influence measures of PRTN3 expression. We measured PRTN3 expression in the spiked samples using RT-dPCR. Predictably, when assessed per unit of starting RNA, the absolute copies of PRTN3 expression reduced as the proportion of fragmented RNA increased (Fig. 2b). Normalisation of PRTN3 expression values effectively corrected for the variable proportion of fragmented RNA, even in samples comprising 50 percent fragmented RNA (Fig. 2b). However, in those samples where the amount of fragmented RNA increased beyond 50 percent, there were indications that normalisation could result in some over-estimation of PRTN3 expression.

### Application of data normalisation to pathological samples

We investigated IL27A gene expression in rheumatoid nodule tissues using dPCR, applying normalisation and appropriate corrections for RNA fragmentation. Within our nodule sample set (n = 27), absolute measures of IL27A expression ranged from 0.25 to 14.16 copies/ng RNA. As a point of reference, a sample returning absolute measures of 0.63 copies of IL27A/ng RNA using dPCR corresponded to an average cycle threshold (C_T_) value of ~37 by qPCR.

**Figure 3:**
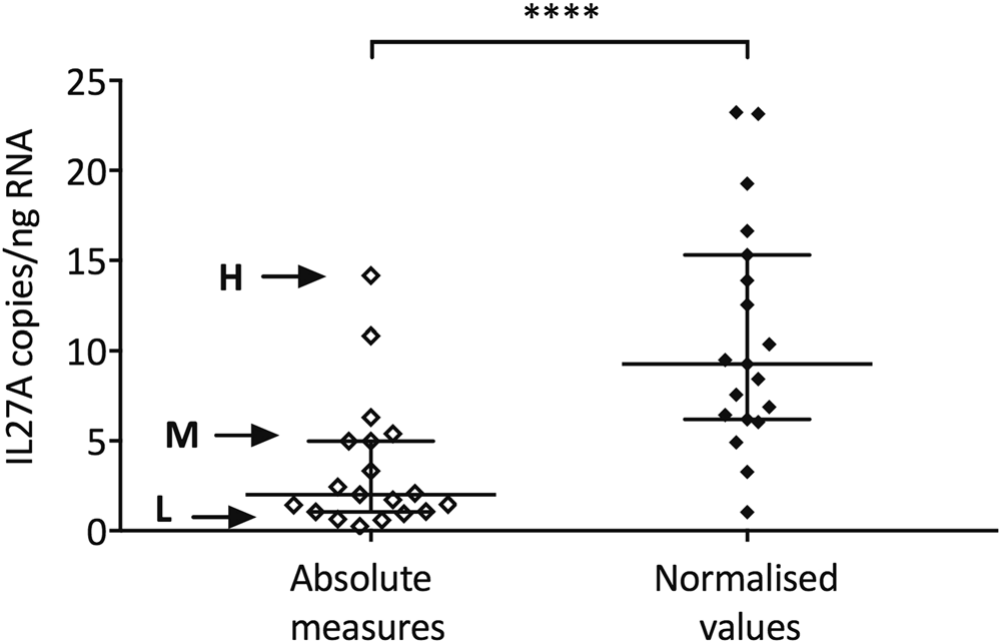
Effect from data normalisation on IL27A expression in rheumatoid nodules. IL27A gene expression in nodule RNA samples (n = 19) measured with dPCR. The data sets are displayed as absolute or normalised values (****p < 0.0001). Three nodule samples spanning the range of dPCR-measured values (H = high; M = medium; L = low) returned corresponding C_T_ values (SD of 3 technical replicates) with qPCR of H: 33.67 (0.297); M: 34.92 (0.149); and L: 37.34 (0.768). Bars indicate median and interquartile ranges.

Based on DV_200_ metrics, nodule samples returning values below 50 percent were then excluded from analysis and we compared absolute measures with normalised values generated from the remaining 19 nodule samples (Fig. 3). Normalisation, resulted in significantly higher median values for IL27A expression (*p* < 0.0001).

## Discussion

Key factors in conducting research in human disease are both the availability and quality of appropriate tissue samples. This combination is often a *fait accompli*, with potential confounders associated with available human tissue, including characteristics of any inherent pathological processes, likely to influence the quality of the tissue and yield of accurate molecular results. This study examined the characteristics of human rheumatoid subcutaneous nodules, an inflammatory lesion with inherent necrosis, associated with granulomatous inflammation. The goal was to identify those features that determine the suitability of variably necrotic tissue for downstream molecular analysis, particularly the impact on the detection of low abundance gene expression using digital PCR.

The critical influence of RNA integrity on downstream molecular analysis is well recognised. Development of measures of RNA integrity from the long-time standard based on 28 S to 18 S ratio, to a predictive model of RNA integrity, the RIN, now provides a much more reliable and robust measure that subsequently correlates with downstream analysis of gene expression^18^. Effectively, measures of RNA integrity generate exclusion criteria that dictate the suitability of samples. The dilemma is the application of these criteria to tissues where there is inherent necrosis as part of the underlying pathological process, which is likely to impact on RNA integrity. In rheumatoid subcutaneous nodules, necrosis is a focus of the inflammatory granuloma. However, tissue necrosis is common during the development of a wide range of diseases, varying in aetiology that includes a “programmed” or “regulated” process, activated in response to death receptor ligands or cellular stress^19–21^, or through a contribution to the cytotoxicity of many chemotherapeutic agents in cancer^10^. In such settings, restricting molecular analysis to high quality tissues limits the available tissue and is unlikely to reflect the true pathological process in operation.

Our results show that despite nodule tissue samples being harvested and treated using optimal RNA-preservation procedures, the presence of inherent, necrotic tissue contributes considerable amounts of highly fragmented RNA. In order to gauge the extent of RNA fragmentation, we considered the DV_200_ metric, indicative of the proportion of extracted RNA transcripts greater than 200 nucleotides in each sample. This metric correlated significantly with expression levels of the endogenous control genes GAPDH and HPRT1, measured by dPCR. This indicates that amongst tissue samples where RNA fragmentation has been naturally generated *in-vivo*, the downstream quantitation of target gene expression predominantly reflects the levels present in that portion of RNA containing the most intact transcripts.

Reference gene normalisation is recommended for gene expression analysis using alternative dPCR approaches, thereby circumventing any impact from RNA sample contaminants^1,22^. Using experimentally generated mixtures of RNA and dPCR assays for proteinase 3 (PRTN3) expression, our results show that normalisation of gene expression data to endogenous control genes’ expression, also accounts for the proportion of intact transcript present amongst highly fragmented RNA. Further, the normalisation of target PRTN3 expression in THP-1 cell RNA, was effective in samples containing as much as 50% fragmented nodule RNA. However, when the proportion of fragmented RNA increased beyond 50%, and up to 75%, normalisation tended to slightly over-estimate target gene expression. Thus, when considerable RNA fragmentation is present, the expression of endogenous control genes is affected and becomes a less accurate reflection of the intact, measurable portion of RNA. Consequently, normalisation becomes less reliable. Our data suggest that implementing a 50% fragmentation threshold, circumvents any problems associated with more fragmented RNA in pathological samples.

We evaluated nodule RNA samples for IL27A gene expression, comparing absolute (non-normalised) and normalised outcomes of dPCR. In these samples, expression of this cytokine gene was generally low by conventional qPCR. Applying the criterion of a DV_200_ metric >50% to ensure reliable normalisation, we excluded 9 of 27 samples from the final data set but subsequently revealed meaningful values for IL27A expression in those remaining. We conclude that normalisation of target gene levels to suitable endogenous controls in RT-dPCR gene expression studies is appropriate when applied to pathological samples where inherent RNA fragmentation is present. The generation of reliable, normalised data also depends on the level of fragmentation within samples, and may require the implementation of appropriate exclusion criteria. In that regard, we do anticipate similar normalisation strategies could be applied when assessing gene expression in formalin-fixed, and paraffin-embedded (FFPE) tissues, where RNA fragmentation and low RIN scores^23^ are an inevitable consequence of the treatment. A more likely factor limiting the assessment of gene expression within FFPE tissues is chemical modification of the RNA, fragmented or not. Consequently, adherence to the recommendations for processing and storage of FFPE tissue that is destined for gene expression analysis^23^ and careful selection and validation of appropriate endogenous control genes^24^ are additional factors for consideration.

## Material and Methods

### Patient demographics and tissue collection

Subcutaneous nodule tissues (n = 27) or joint synovial tissues (n = 27) were obtained from patients (n = 42) with rheumatoid arthritis (RA), as defined by 1987 American Rheumatism Association Classification criteria^25^, undergoing elective surgery for nodule removal. The patient cohort comprised those of age 64 ± 9 years (mean ± SE), disease duration 21 ± 3 years, with the majority positive for both rheumatoid facter and anti-citrullinated peptide antibodies. Five patients provided multiple nodules, and 3 further patients multiple synovia, either at the same or different times. The study was approved by the New Zealand Health and Disability Multi-Regional Ethics Committee (MEC06/02/003). All patients provided informed written consent prior to tissue donation and all experiments were performed in accordance with relevant guidelines and regulations.

Nodule and synovial membrane tissues were processed immediately following surgical excision in an RNase-free environment or collected into RNAlater (Invitrogen) at 4 °C. Apportioned tissues destined for gene expression studies were snap-frozen and stored in liquid nitrogen.

### RNA extraction, analysis and reverse transcription

The RNeasy Mini Kit (Qiagen) was used according to manufacturers’ recommendations, with DNase digestion either on-column using the RNase-free DNase Set (Qiagen) or post-extraction with PerfeCTa DNase I, (Quantabio). Eluted RNA concentration was determined using a Qubit Fluorometer (Invitrogen) with low or high sensitivity Qubit assay kit (Molecular Probes). Up to 1000ng of total RNA was added per 20ul reaction with Superscript III Reverse Transcriptase (Invitrogen) and primed with Oligo (dT)_20_ (Invitrogen) as per the manufacturer’s protocol to generate cDNA. RNA fragment and DV_200_ metric analysis were conducted by NZ Genomics Ltd using an Illumina Fragment analyser or Agilent BioAnalyzer and associated software.

### Digital PCR

Digital PCR (dPCR) was performed using the Quantstudio 3D Digital PCR system (Applied Biosystems) including the chip loader, ProFlex thermal cycler, and chip reader. Reactions were prepared in 15ul volumes with Quantstudio 3D Digital PCR Mastermix v2 (Applied Biosystems), Taqman gene expression assays (IL27A: Hs00377366_m1, VIC; GAPDH: Hs99999905_m1, FAM at 0.5X concentration in duplex with HPRT1: Hs99999909_m1, VIC; PRTN3: Hs01553330_m1, FAM; Applied Biosystems), and appropriate sample input amounts (GAPDH/HPRT1 duplex: cDNA representing 0.25 ng/ul RNA; IL27A: cDNA representing 5 ng/ul RNA; PRTN3: cDNA representing 3.125 pg/ul RNA). Reactions were loaded onto Quantstudio 3D 20 K V2 dPCR chips (Applied Biosystems) and cycled as follows: PRTN3 10 min at 96 °C, 39X (2 min at 55 °C, 30 sec at 98 °C), 2 min at 55 °C, held at 10 °C; all remaining assays: 10 min at 96 °C, 39X (2 min at 60 °C, 30 sec at 98 °C), 2 min at 60 °C, held at 10 °C. Prior to running duplex reactions, assays were validated to ensure the duplex readings were consistent with respective single-plex assay readings over a range of input cDNA concentrations (data not shown). Quantstudio 3D AnalysisSuite Cloud Software (Applied Biosystems, version 3.1.2-PRC-build-03) with Poisson Plus quantification algorithm (version 4.4.10) and 95% CI was used. Imported eds files were individually assessed and fluorescence thresholds set manually to generate transcript copies/ul, then converted to copies/unit RNA.

To achieve data normalisation, copies/unit RNA of the target gene were multiplied by the corresponding normalisation factor (geometric mean of GAPDH and HPRT1 normalisation factors) and final data displayed as normalised copies/unit of RNA. The stable expression of the two endogenous control genes was previously assessed among equivalent nodule samples using qPCR with qbasePLUS v2.1 (Biogazelle)^26^. An average coefficient of variation (CV) of 23.3%, and M value of 0.658 for the two genes was generated.

### Validation Study

Available data from RNA-seq analysis identified high expression of the proteinase 3 gene (PRTN3) in unstimulated THP-1 cells (mean FPKM = 2262, n = 3). In comparison, PRTN3 expression within nodule tissues was low (mean FPKM = 0.2948; n = 8; unpublished observations). This contrast in expression was used to validate the use of normalisation to account for variable fragmentation within sample RNA. We pooled naturally, highly fragmented RNA (RIN = 2.5; DV_200_ = 10%) from 4 rheumatoid nodules, then generated a series of samples containing set proportions (100–0%) of good quality THP-1 cell RNA (RIN = 8.3; DV_200_ = 93%), spiked with increasing proportions of the pooled, highly fragmented nodule RNA (0–100%). This nodule RNA contained negligible values for averaged GAPDH and HPRT1 expression (0.0017 copies/pg RNA).

In each sample, RNA quality parameters were assessed by NZ Genomics using an Agilent BioAnalyzer. Two hundred and fifty ng of total RNA from each prepared sample, verified by Qubit fluorometry (Invitrogen), was reverse transcribed and expression of GAPDH and HPRT1 endogenous control genes and the PRTN3 target gene measured with RT-dPCR.

### Quantitative real-time PCR

Samples identified as having high, medium or low IL27A expression levels by dPCR were compared for IL27A expression in routine qPCR. Ten uL reactions containing Quanta ToughMix (Quanta Biosciences), Taqman Assay (Hs00377366_m1 (VIC)), and cDNA representing 31.25ng starting RNA, were assayed in triplicate using a Roche LC480 (Roche Molecular Systems) and standard cycling and detection protocols.

### Statistics

Graphpad Prism V7.0b (Graphpad Software) was used for statistical analyses. An unparied t test with Welch’s correction for unequal variance was used to compare the DV_200_ metrics between 27 nodules and 27 synovia, following a D’Agostino & Pearson test establishing normal distribution (p > 0.05). The geometric mean of the GAPDH and HPRT1 expression was correlated with the associated DV_200_ metric values using a two-tailed pearson correlation test with 95% CI for log-transformed nodule data (n = 27) and spiked samples (n = 5) (Shapiro-Wilk normality test p > 0.05). Absolute and normalised IL27A gene expression values were compared using a two-tailed non-parametric Mann-Whitney test, following assessment with D’Agostino & Pearson normalisty test (p < 0.05, n = 20).

### Data availability

Nodule RNAseq datasets referred to during the current study are not yet publicly available, as complete data analysis is part of on-going research. Information therein is available from the corrsponding author on reasonable request.

## Electronic supplementary material

Supplementary Figures
