# Supplementary material for: Digital-PCR for gene expression: impact from inherent tissue RNA degradation
Source: Sci Rep. 2017 Dec 8;7:17235. doi: 10.1038/s41598-017-17619-0 (PMC5722939; doi:10.1038/s41598-017-17619-0)
Supplement: Supplementary file 1 — Supplementary Figures [file 41598_2017_17619_MOESM1_ESM.pdf]

Digital-PCR for gene expression: impact from inherent tissue RNA degradation

Melanie J. Millier, Lisa K. Stamp, Paul A. Hessian

Supplementary Information

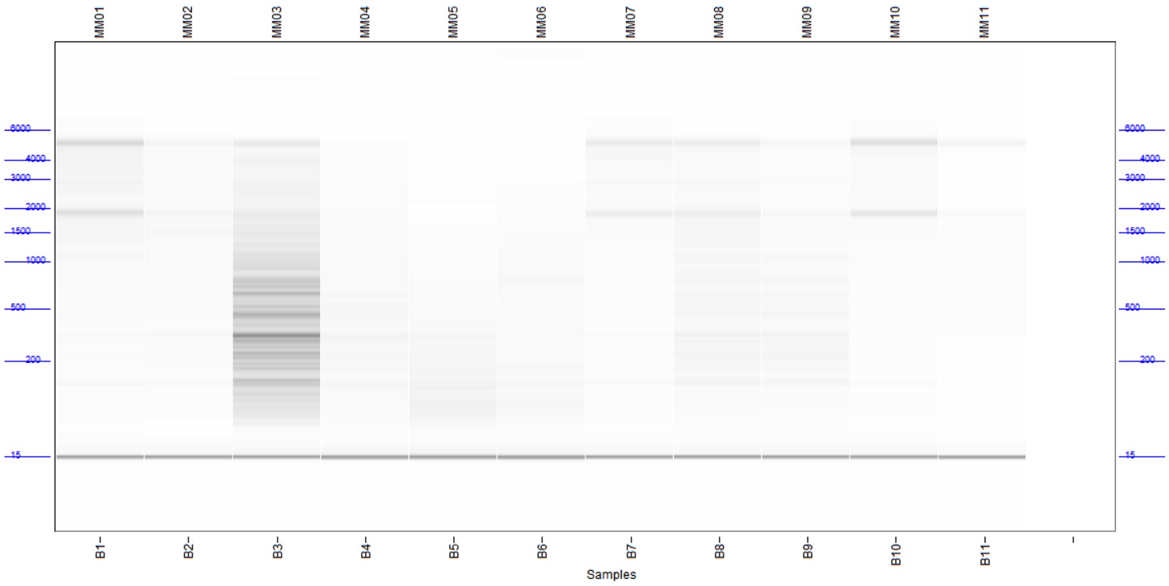

Supplementary Figure S1. Capillary electrophoresis gel images of subcutaneous nodule RNA.

Figure is the original pdf results file showing capillary electrophoresis gel images for 11 separate nodule RNA samples. Samples B10, B8 and B5 correspond to nodule samples 1, 2 and 3 respectively, shown in figure 1(a) of the manuscript. For publication, this entire figure was contrast enhanced to highlight fragmented RNA, then appropriately cropped and uniformly resized.

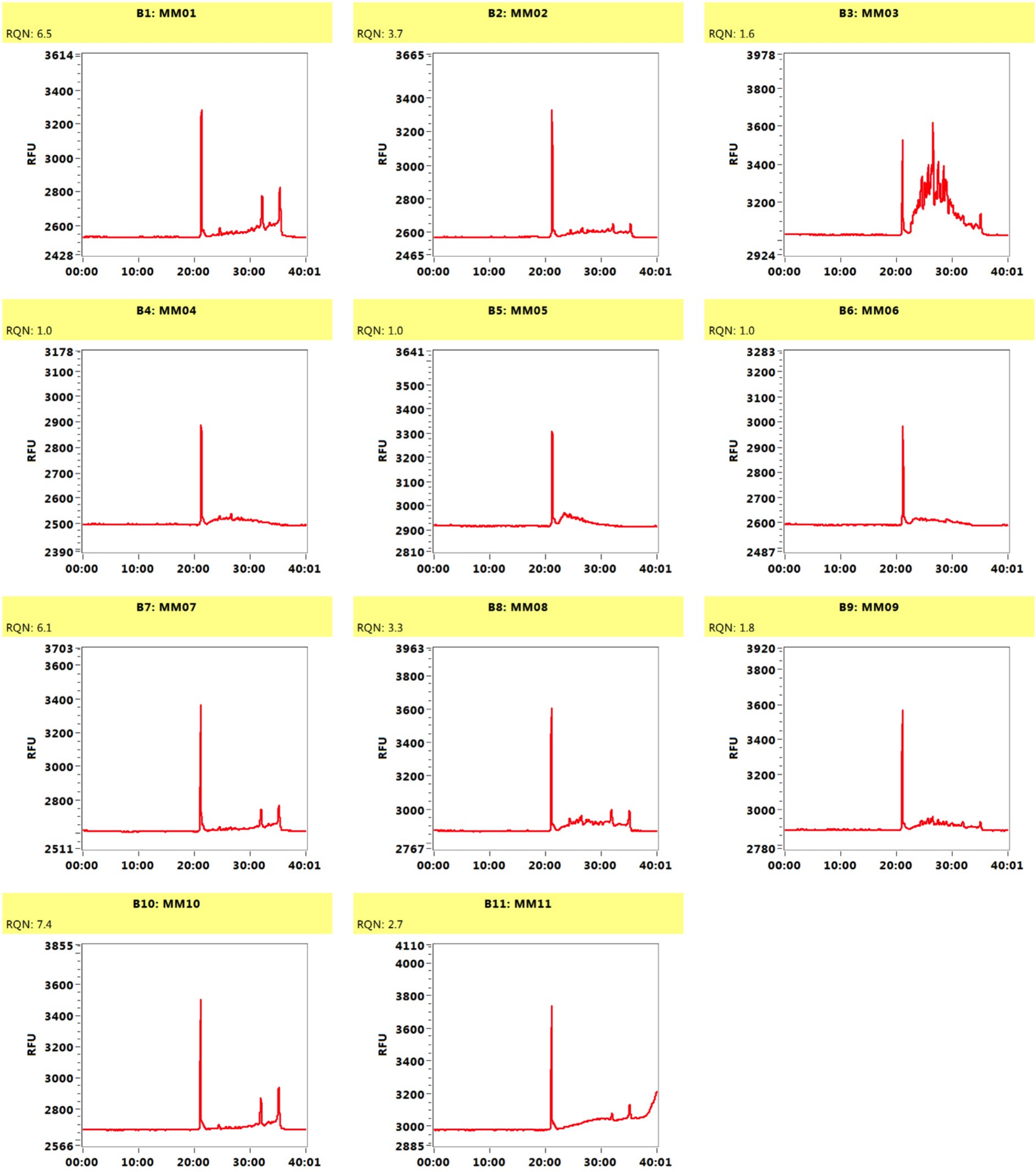

Figure S2. Electropherograms of subcutaneous nodule RNA

Figure is the original pdf results file showing individual electropherograms for 11 separate nodule RNA samples. The electropherograms B10, B8 and B5 correspond to those for nodule samples 1, 2 and 3 shown as black and white versions in figure 1(a) of the manuscript.
